# Hypocalcemia-Induced Seizure: Demystifying the Calcium Paradox

**DOI:** 10.1177/1759091415578050

**Published:** 2015-03-23

**Authors:** Pengcheng Han, Bradley J. Trinidad, Jiong Shi

**Affiliations:** 1 Barrow Neurological Institute, Dignity Health St Joseph's Hospital and Medical Center and Medical Center, Phoenix, AZ, USA; 2 Creighton University School of Medicine—Phoenix Campus, Phoenix, AZ, USA

**Keywords:** action potential, hypocalcemia, ion channel, neurophysiology, seizure, synaptic transmission

## Abstract

Calcium is essential for both neurotransmitter release and muscle contraction. Given these important physiological processes, it seems reasonable to assume that hypocalcemia may lead to reduced neuromuscular excitability. Counterintuitively, however, clinical observation has frequently documented hypocalcemia’s role in induction of seizures and general excitability processes such as tetany, Chvostek’s sign, and bronchospasm. The mechanism of this calcium paradox remains elusive, and very few pathophysiological studies have addressed this conundrum. Nevertheless, several studies primarily addressing other biophysical issues have provided some clues. In this review, we analyze the data of these studies and propose an integrative model to explain this hypocalcemic paradox.

## Introduction

There are many common neurological manifestations of hypocalcemia including tetany, seizure, and delirium, suggesting a role for hypocalcemia in increasing excitability in the central nervous system. Chvostek’s sign is a sensitive clinical indication of hypocalcemia, manifesting as hyperexcitability in the nerve endings of facial muscles. Hypocalcemia also frequently accompanies colicky gastrointestinal symptoms, suggesting hyperexcitability in the vagal nerve system. These are some of the few examples of how hypocalcemia universally enhances neuromuscular excitability throughout the body.

Hypocalcemia-induced seizures, in particular, have attracted much clinical attention. These seizures likely occur in patients with predisposing endocrinological abnormalities or renal insufficiency with overall poor calcium homeostasis. For example, a seizure could be the first manifestation of chromosome22q syndrome (Tsai et al., 2009; Kinoshita et al., 2010) due to low blood calcium as a result of congenital hypoparathyroidism. Another example of hypocalcemic seizures, and perhaps the most commonly seen, are drug-induced hypocalcemic seizures (Milman and Epstein, 2010). Biphosphonate may also induce hypocalcemic seizures due to a disturbance of calcium and phosphate metabolism (Maclsaac et al., 2002; Tsourdi et al., 2011). Finally, the anticonvulsant phenytoin may paradoxically exacerbate seizures when blood calcium is low (Ali et al., 2004). Therefore, clinicians are advised to check calcium in cases of Anti-epileptic drug (AED) refractory seizures.

Although hypocalcemic seizures are widely documented in medical literature (Erdeve et al., 2007; Tsai et al., 2009; Milman and Epstein, 2010; El Asri et al., 2012; Kidwell et al., 2013; Korkmaz, et al., 2014), the underlying mechanism seems counterintuitive. From the presynaptic release of neurotransmitters, to the electrical mechanical coupling in the myocyte, almost every step in the neuromuscular function is predicated on calcium (Sudhof, 2013; Baylor and Hollingworth, 2010; Lazarevic et al., 2013). However, clinical observations clearly demonstrate an inverse relationship between excitability and blood calcium: Hypocalcemia enhances neuronal excitability whereas hypercalcemia decreases excitability. This paradox has not been adequately studied and explained. Nevertheless, several published studies, primarily aimed at addressing other topics, have revealed a few clues. Here, we critically analyze this “side data” and piece together these clues, aiming to understand the mechanism of hypocalcemic seizures and associated hyperexcitable symptoms. We keep in mind readers who may have less background on ion channel physiology. For epilepsy specialists or neurophysiologists, we recommend skipping some of the background information.

## External Calcium Versus Internal Calcium

The calcium paradox described above partially arises from the concept of extracellular (external) calcium and intracellular (internal) calcium. Most medical literature prefer to use extracellular or intracellular to emphasize the anatomical concept. The electrophysiological literature conventionally use external and internal as they refer to the preparation of experimental bath solution or pipette solution. In this review, we use this nomenclature interchangeably.

The external calcium represents blood calcium, where the physiological range of total calcium is in the range of 2.2 to 2.6 mM. Approximately half is protein bound and the other half is free and functionally active (Morton et al., 2010). A reasonable estimation would be 1.0 to 1.3 mM free calcium bathing the cells outside the cell membrane. In contrast, internal calcium can be divided into two compartments: internal calcium stores and calcium in cell plasma. Cell plasma calcium is estimated to be in the range of nanomoles but could rise to micromoles in the proximity of voltage-gated calcium channels (VGCC; Neher and Sakaba, 2008).

The external calcium concentration is relatively constant unless affected by a significant organ malfunction such as severe renal function impairment, tumor lysis syndrome, hyperparathyroidism, hypoparathyroidism, or severe insufficient vitamin D or calcium intake. The cell plasma calcium is determined by calcium influx from the external compartment and by calcium release from internal stores. While intracellular calcium has been widely studied due to its crucial role as a second messenger in cell signaling, the effects of extracellular calcium have not been as thoroughly investigated. The external calcium may have direct and distinct physiological roles apart from replenishing the internal calcium.

Ultimately, any change of the neuronal excitability could be attributed to one or more ion channel families that determine the properties of action potential firing. Most of these channels are subject to Calmodulin (CaM) modulation on the intracellular side (Zuhlke et al., 2000). There is no doubt that intracellular calcium modulates a variety of ion channels. Here, the key question is how the external calcium modulates these candidate ion channels. The extracellular calcium modulates their target ion channels through three possible pathways.

First, external calcium directly binds to the extracellular domain of their target ion channels and allosterically modulates their gating behavior. This is the most simple and straightforward pathway. Second, external calcium can influx into the cells through various calcium-permeable channels such as VGCC, N-methyl-D-aspartate channels, and acetylcholine nicotinic receptors. Calcium influx transiently increases the internal calcium, which in turn modulates other ion channels from inside. Third, external calcium activates calcium sensing receptors (CaSR). CaSR were initially studied in the parathyroid gland, a key organ maintaining blood calcium homeostasis (Brown et al., 1993). CaSR transcripts were subsequently identified in neurons and glia (Yano et al., 2004). CaSR are G-protein-coupled receptors linking to intracellular secondary signals including the phospholipase C (PLC) and cyclic adenosine monophosphate (cAMP) pathways. By activating CaSR without necessarily entering the cells, external calcium indirectly mobilizes intracellular secondary signaling molecules to modulate multiple targets including ion channels (Bouschet and Henley, 2005).

In the following sections, we analyze available studies addressing the multiple effects of external calcium on action potential parameters. Guided by this information, we then further analyze the relevant ion channels individually. We describe which one of the three modulatory pathways is involved in each individual type of ion channels.

## The Effects of External Calcium on Neuronal Action Potentials

When rat hippocampal slices were incubated in a low calcium solution for a prolonged duration of at least 60 min, spontaneous epileptiform bursting appears on the extracellular field potential recording (Roper et al., 1992, 1993; Richardson and O’Reilly, 1995; Ghai et al., 2000). This bursting is not dependent on synaptic transmission because excitatory transmission blockers (AP-5/DNQX) had been included in the bath solution (Roper et al., 1992). As such, it was described as “nonsynaptic epileptiform activity,” suggesting a change of intrinsic properties of individual neurons independent of network activity. Indeed, low calcium bursting was confirmed by intracellular recording showing an enhanced frequency of action potential (Bikson et al., 2002). Wang et al. examined the effects of calcium and magnesium on burst firing and excitability in hippocampal neurons (Wang et al., 2004). Given the same magnesium concentration (2 mM), a reduction of external calcium from 2 to 1 mM increased action potential burst frequency from 28 to 171 Hz, a dramatic sixfold increase. This dramatic firing rate could be partially explained by an increase in resting membrane potential and a decrease of firing threshold. The resting membrane potential was increased from −67.5 to −64 mV. The firing threshold was decreased from −59.3 to −63.3 mV. However, Wang et al. did not provide any further analysis on the ion channel mechanism that affects these intrinsic firing properties. In our opinion, although both the elevation of resting membrane potential and the suppression of thresholds are small, they draw close the distance between the resting potential and the threshold. As the external calcium concentration is halved, threshold potential (−63.3 mV) almost equals the resting membrane potential (−64 mV), thus making the cells very likely to be excited. In agreement with this hypothesis, reducing external Ca^2+^ from 2 to 1 mM resulted in a 100% appearance of recurrent or continuous seizure-like activity (Isaev et al., 2012). Reduction of Ca^2+^ to 1 mM is identical to an increase of external K^+^ to 5 mM in terms of changes in cellular excitability and seizure threshold. An increase of Ca^2+^ to 3 mM completely abolished seizure-like activity generation even in the presence of high K^+^ (Isaev et al., 2012). These studies suggest that external calcium may modulate groups of voltage-gated ion channels and therefore change at least three intrinsic firing properties: resting membrane potential, firing threshold, and action potential frequency.

## Candidate Ion Channels Determining Resting Membrane Potential

A hyperpolarized resting membrane potential silences cells while depolarization increases cell excitability. For the most part, the resting membrane potential is determined by the potassium gradient across the cell membrane. The K^+^ flux across the membrane under resting conditions occurs through the K^+^ leak channel, a two-pore K^+^ channel family (K2P channel). Assuming external K^+^ is 4 mM and internal K^+^ is 130 mM, the K^+^ reversal potential is −93 mV as predicted by the Nernst equation. Measured neuronal resting membrane potential is approximately −65 to −75 mV. This 20 mV difference between K^+^ reversal potential and resting membrane potential is contributed by a small conducting Na^+^ leak channel (NALCN), which gives a depolarizing current (DC; Gilon and Rorsman, 2009). Notably, this NALCN is not the voltage-gated sodium channel (NaV) that initiates an action potential. We discuss the NaV in the next section. It is fair to say that the resting membrane potential is mostly determined by K^+^ leak channels, with NALCNs playing a minor but nonnegligible role.

Whether external calcium has an effect on the K^+^ leak channel is not known. However, external calcium does shut the gating of the NALCN and reduces Na^+^ current. Lu et al. proposed a model linking external calcium and the NALCN via the CaSR, activation of G-protein, and the UNC80/UNC79 protein complex (Lu et al., 2010). According to this model, high calcium activates the CaSR, which tonically inhibits the NALCN. Reducing external calcium relieves this inhibition and favors the depolarizing Na^+^ leak current. This model nicely explains the upshift of the resting membrane potential toward the firing threshold when the external calcium is low.

## Ion Channels Relating to Firing Threshold

### Voltage-Gated Na^+^ Channel

The voltage dependency of NaV activation is the key factor that determines the threshold voltage to fire an action potential. Hille discovered that high calcium shifted the voltage-dependency of sodium channel activation gates to the right, suggesting that a higher depolarization is necessary to open NaV (Hille, 1968). Conversely, low calcium makes the sodium channel open at a relatively negative voltage. Hille attributed this effect to a simple surface charge. According to this surface charge hypothesis, as more calcium binds to the outer surface, the transmembranous electric field takes on a more hyperpolarized nature. For example, adding positive-charged calcium ions to a −70 mV transmembrane potential may perhaps bring the apparent electric field down to −80 mV, therefore making the ion channels located across the cell membrane “feel like” they are in an ambient electric field of −80 mV. Therefore, these channels need to overcome a larger hurdle to activate. According to this concise and appealing model, Mg^2+^, which has the same charge quantity as Ca^2+^, should affect the firing threshold to the same degree. Experimental evidence suggests that reducing Mg^2+^ concentration decreases neuronal firing threshold as well but to a much less extent compared with Ca^2+^ reduction (Wang et al., 2004). The additional outer surface charge brought by high external calcium would provide an equal electric field for both Na^+^ and K^+^ channels. However, Hille found that external calcium had no modulatory effect on delayed rectifier potassium channels (Kdr). Therefore, the surface charge model remains insufficient to explain the underlying mechanism (Hille, 1968).

### Voltage-Gated K^+^ Channel

As demonstrated by Hille, changing external calcium concentration had no effect on delayed rectifier K^+^ channel (Kdr) activation. Inward rectifier K^+^ channel (Kir), however, decreased input resistance of myenteric neurons at resting potential and hence reduced the excitability (Rugiero et al. 2002). Because the Kir is only weakly inhibited by external calcium (Wegner, 1994), the contribution of Kir modulation in the situation of low external calcium may be small, if any.

Transient K^+^ current is a unique voltage-dependent outward current because it spontaneously inactivates several milliseconds after channel activation. Due to its current trace resemble letter “A,” it is usually referred to as “A-type current (I_A_)” (Birnbaum et al., 2004). I_A_ provides a resistant force to hinder the start of action potentials (Han and Lucero, 2005). Reducing I_A_ will reduce the firing threshold (Han et al., 2007). It is unknown whether external calcium modulates I_A_ in mammalian cells. Nevertheless, in drosophila, neuronal I_A_ current amplitude increased by 109% when the external calcium was switched from 0 to 5 mM. Meanwhile, I_A_ inactivation voltage dependency shifted toward the right, suggesting that I_A_ current is less likely to inactivate at a higher concentration of external calcium (Xu et al., 2005). Thus, higher calcium enhances I_A_ amplitude and removes I_A_ inactivation, which may contribute to a transient shunting of current, leading to a higher firing threshold. Conversely, low calcium depresses I_A_ and hence favors a low firing threshold.

## Ion Channels Relating to Firing Frequency

### Calcium-Activated Potassium Channel (KCa Channel)

**Figure 1.**
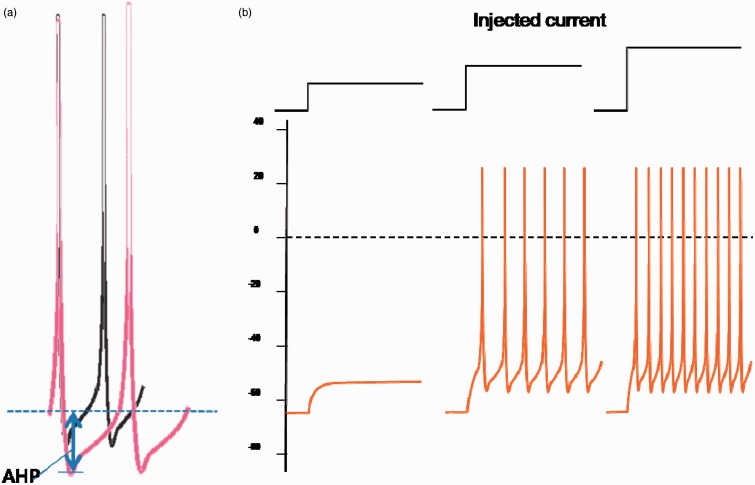
Modulation of firing frequency. (a) Afterhyperpolarization determines the interspike interval. (b) Injected depolarizing current enhances firing frequency. Figures modified from Han et al. (2007). AHP = afterhyperpolarization.

The interval between adjacent action potential spikes (interspike interval) is a key factor that determines firing frequency (Han et al., 2007). After the membrane potential repolarizes back to resting level, a further transient hyperpolarization may be present after the end of the action potential. This hyperpolarization valley is named the afterhyperpolarization (AHP, Figure 1a). It is the valley’s depth (AHP amplitude) that determines the time interval before reaching back to the next firing threshold. A basic rule of thumb is that a small AHP translates into a higher firing frequency while a larger AHP will reduce firing frequency. While multiple types of potassium channels contribute to AHP, it is the KCa channels that may fine tune the actual depth of the AHP (Sah, 1996). KCa channels have an intracellular domain that binds to intracellular calcium. High internal Ca^2+^ enhances K^+^ outflow thus increasing the AHP and slowing the firing rate. This suggests a reasonable model that action potentials open VGCC leading to a transient increase in cytoplasmic Ca, which in turn modulates these KCa. Conversely, low external calcium would provide less calcium influx and diminish KCa activation.

In dorsal root ganglion neurons, decreasing the external calcium concentration from 1 to 0.35 mM reduced calcium current by 6%, whereas increasing external calcium to 7 mM enhanced calcium current by 35% (Hogan et al., 2008). Furthermore, decreasing calcium current significantly reduced AHP. These data confirmed the hypothesized sequential model: High external calcium provides a strong calcium influx through calcium channels, and the incoming calcium elevates the internal calcium, in turn activating KCa. K^+^ flows through these KCa, subsequently leading to hyperpolarization.

However, modulation of the KCa may not rely on calcium influx but instead on CaSR (Vassilev et al., 1997). Using single channel recording, Vassilev et al. recorded intermediate conductance of KCa current from hippocampal neurons. Increasing external calcium from 0.5 to 3 mM led to significantly increased KCa amplitude. This KCa enhancement was not observed in CaSR knockout mice. Interestingly, neomycine potentially activates the CaSR and enhances KCa. The same research group also reported similar CaSR modulation of KCa in oligodentrocytes (Chattopadhyay et al., 1998). These data suggest an alternative sequential model: external calcium—>CaSR—>G-protein—>KCa.

Taken together, external calcium enhances KCa in both peripheral and central neurons, thereby potentiating AHP and reducing neuronal firing frequency. With regard to the question of how external calcium targets to KCa in the intracellular modulatory domain, it is theorized that calcium may act through CaSR without physically entering the cell.

### Cyclic Nucleotide-Gated Channel

Besides AHP, firing frequency is also determined by injected positive DC. During intracellular recording, a stepwise DC was conventionally injected to model various levels of excitatory inputs. The firing frequency increased with the injected DC (Figure 1b). In hippocampal CA1 neurons, activating acetylcholine M1/M3 receptors induced a constant lasting DC, which translated into a depolarizing voltage plateau (Kuzmiski and MacVicar, 2001). This depolarization may favor epileptic firing. Acetylcholine M1/M3 receptors induce an increase in cyclic guanosine monophosphate (cGMP), which in turn opens cyclic nucleotide-gated ion channels (CNG). The influx of Na^+^ and Ca^2+^ through CNG channels contributes to the depolarizing plateau (Kuzmiski and MacVicar, 2001).

CNG channels were first identified in retina and olfactory receptor neurons (Biel and Michalakis, 2009). CNG channels are nonselective for monovalent or divalent ions that compete for the channel pore. Divalent ions contribute less current but have higher affinity to the pore due to their relatively heavier positive charges. Thus, removing calcium facilitates sodium entry through CNG channels, providing a larger CNG current. Increasing extracellular Ca^2+^ decreased the total CNG current (Kleene and Pun, 1996). Therefore, lowering calcium may enhance CNG current and thus increase proseizure plateau current in hippocampal neurons.

## The Effect of External Calcium on Ligand-Gated Channels

### Alpha-amino-3-dyroxy-5-methyl-4-isoxazolepropionic acid (AMPA) Receptor Channel

When AMPA channels in the central nervous system receive an excitatory response from glutamate transmitters, inward cationic current rushes through these channels providing the initial depolarization for eventual signal. Work by Dreixler and Leonard attempted to highlight the role of calcium, along with another ion—zinc—in AMPA current potentiation. To understand zinc’s role, Dreixler and Leonard measured the current flowing through AMPA channels reconstructed in xenopus oocytes (Dreixler and Leonard, 1997). For both GluR1 and GluR3, high zinc concentration inhibited AMPA current; modest concentration (less than 50 µM), however, did not change the current amplitude, provided that physiological ranges of calcium (1.8 mM) were included in the extracellular solution. When the extracellular solution was replaced with calcium free solution, zinc strongly increased the AMPA current. When zinc concentration was kept constant, increasing calcium concentration progressively reduced AMPA current. This observation suggests that external calcium may compete with zinc binding sites on AMPA receptors, and thus high calcium concentrations may inhibit zinc potentiation of AMPA current, whereas low or zero calcium leaves greater freedom for zinc to bind and enhance the AMPA current.

### Gamma-aminobutyric acid (GABA)

GABA-a receptor is a ligand-gated chloride channel that provides either hyperpolarization or shunting effects. GABA-a receptors are the molecular base for inhibitory postsynaptic current. The GABA-b receptor is a metabotrophic receptor that enhances G protein-coupled inwardly-rectifying potassium channel (GIRK) channels, which provide a tonic hyperpolarizing current (Mutneja et al., 2005; Hearing et al., 2013).

There is no direct examination of the effects of external calcium on GABA-Cl^−^ current. An indirect clue from Llano et al. (1991) showed Ca^2+^ entry induced by depolarizing voltage pulses potentiated GABA-Cl^−^ current. However, the same depolarizing pulse inhibits spontaneous inhibitory synaptic current. Thus, we are unable to postulate the effect of external calcium on the GABA receptor.

Wise et al. showed that relatively high external calcium (1 mM) potentiates GABA-b receptors via CaSR, while relatively low calcium (0.01 mM) inhibits them (Wise et al., 1999). However, even the highest calcium concentration in this study (1 mM) remains to be considered hypocalcemic by clinical standards. The external calcium modulation of GABA-b would be established only if the experimental calcium concentration could have been extrapolated to the range of 1 to 3 mM.

## The Effect of External Calcium on Neurotransmitter Release

Neuronal excitability not only depends on intrinsic ion channels but is also affected by presynatic input from upstream neurons. Patch clamp recording from paired cortical neurons showed that CaSR depressed evoked excitatory postsynaptic current by blocking nonselective cation channels (NSCC) located in the presynaptic nerve terminals (Phillips et al., 2008). NSCCs provide a background depolarization so to speak, to facilitate neurotransmitter release. High external calcium or CaSR agonists (i.e., spermine) overstimulated CaSR and reduced the release of glutamate. Reducing external calcium or knocking out CaSR significantly enhances excitatory postsynaptic currents. It is not clear if the same modulatory mechanism applies to inhibitory presynaptic terminals. If so, the ultimate effect on postsynaptic neurons will be quite complicated, depending on the relative weight of excitatory versus inhibitory inputs.

## Summary

**Figure 2.**
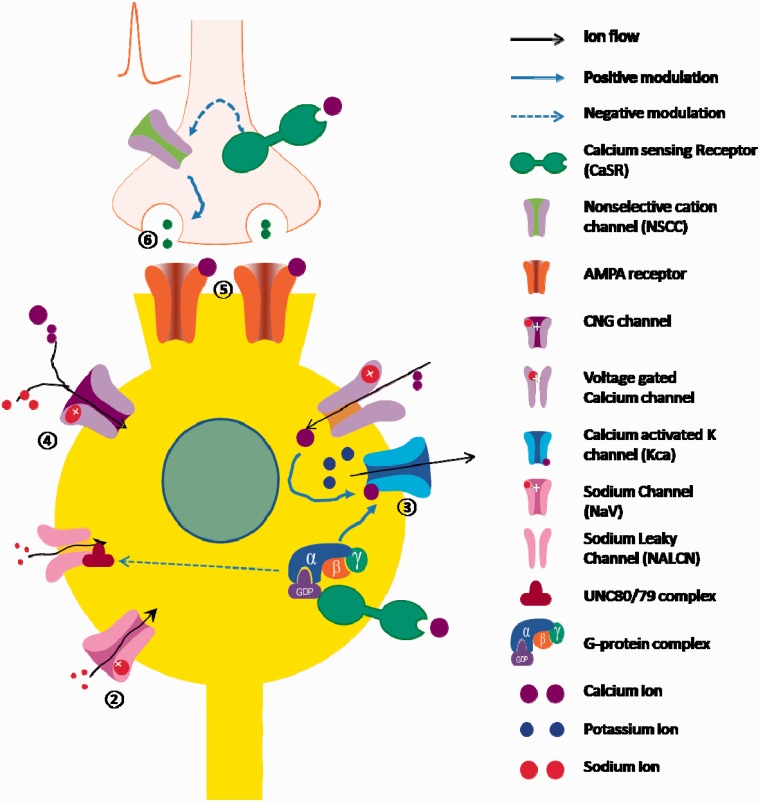
Summary of the target ion channels modulated by external calcium. (1) By activating calcium sensing receptors signaling, external calcium reduces sodium influx through Na^+^ leaky channel , whereas low calcium enhances sodium influx; (2) high external calcium reduces sodium influx through voltage-gated sodium channels, whereas low external calcium enhances sodium influx through a proposed “surface charge” mechanism; (3) high external calcium enhances potassium outflow, whereas low external calcium reduces potassium outflow; (4) high external calcium reduces cation influx, whereas low external calcium enhances cation influx through cyclic nucleotide-gated ion channels; (5) high external calcium reduces ion influx through AMPA receptors, whereas low external calcium enhances it; (6) by activating calcium sensing receptors, external calcium inhibits glutamate release, whereas low external calcium enhances it.

The inverse relationship between extracellular calcium and neuronal excitability could be explained by several complementary molecular events (summarized in Table 1 and Figure 2). External calcium inhibits NALCNs, shifts the voltage dependency of voltage-gated Na^+^ channels, stabilizes CNG channels, reduces inward current through AMPA channels, and depresses the release of excitatory neurotransmitters. Conversely, it enhances transient K^+^ current and KCa channels and perhaps potentiates GABA sensitivity. Some of these modulatory effects may depend on CaSR while others may require calcium influx. It is these processes that we theorize may help shed light on the calcium paradox and lead to further understanding of the mechanisms behind production of seizures through hypocalcemia.

**Table 1.** The Effects of External Calcium on Multiple Ion Channels or Targeted Molecules.

Ion channels/targeted molecules	High external calcium	Low external calcium	Proposed mechanisms
NALCN	↓	↑	CaSR
NaV	↓	↑	Surface charge
Kdr	–	–	
A-type K^+^ current	↑	↓	Unknown
KCa	↑	↓	CaSR or Ca^2+^ influx
CNG channel	↓	↑	Ca^2+^ and Na^+^ competition
AMPA receptor/channel	↓	↑	Unknown
GABA receptor/channel	↑?	↓?	Only indirect evidence, mechanism unknown
EPSC/glutamate release	↓	↑	CaSR

*Note.* NALCN = Na^+^ leaky channel; voltage-gated sodium channel = voltage-gated sodium channel; KCa = calcium-activated potassium channel; CNG = cyclic nucleotide-gated ion channels; EPSC = excitatory postsynaptic current; CaSR = calcium sensing receptors.

We cannot conclude without cautions. First, our model is based on fragmental clues from different cells and species. The numerical definition of “high” or “low” calcium was not uniform among these studies. The range of external calcium reported in these studies varies from 0 to 5 mM. We did not include it in our analysis if the tested calcium concentration was beyond 5 mM due to its irrelevance in the clinical setting. Even the range of 0 to 5 mM is quite extreme and may not represent common clinical situations. Second, the Mg^2+^ concentration varies among these studies. The interaction of Mg^2+^ and Ca^2+^ may constitute a confounding factor. Nevertheless, these studies showed a consistent trend that relatively high calcium reduces excitability and relatively low calcium increases cell firing. This knowledge provides a unifying explanation for the role of hypocalcemic hyperexcitability in seizure activity.
